# Sodium nitrate decreases agrin-induced acetylcholine receptor clustering

**DOI:** 10.1186/s40360-016-0062-0

**Published:** 2016-05-01

**Authors:** Jess Jarosz, Cullen White, Wade A. Grow

**Affiliations:** Department of Anatomy, Midwestern University, 19555 N. 59th Avenue, Glendale, AZ 85308 USA

**Keywords:** AChR, Agrin, C2C12, Myogenin, Sodium nitrate

## Abstract

**Background:**

Humans are exposed to nitrate predominantly through diet with peak plasma concentrations within an hour after ingestion, but additional exposure is obtained from the environment, and minimally through *de novo* synthesis. Higher nitrate consumption has been associated with methemoglobinemia, spontaneous abortions, atherosclerosis, myocardial ischemia, septic and distressed lung, inflammatory bowel disease, amyotrophic lateral sclerosis, and neural tube defects. However, skeletal muscle development has not been examined.

**Methods:**

C2C12 skeletal muscle cell cultures were maintained, myoblasts were fused into myotubes, and then cultures were exposed to motor neuron derived agrin to enhance acetylcholine receptor (AChR) clustering. Untreated cultures were compared with cultures exposed to sodium nitrate at concentrations ranging from 10 ng/mL–100 μg/mL.

**Results:**

The results reported here demonstrate that 1 μg/mL sodium nitrate was sufficient to decrease the frequency of agrin-induced AChR clustering without affecting myotube formation. In addition, concentrations of sodium nitrate of 1 μg/mL or 100 μg/mL decreased gene expression of the myogenic transcription factor myogenin and AChR in correlation with the agrin-induced AChR clustering data.

**Conclusions:**

These results reveal that sodium nitrate decreases the frequency of agrin-induced AChR clustering by a mechanism that includes myogenin and AChR gene expression. As a consequence sodium nitrate may pose a risk for skeletal muscle development and subsequent neuromuscular synapse formation in humans.

## Background

For over 70 years methemoglobinemia in infants has been linked to nitrate consumption [1, 2]. Normally a small percentage of hemoglobin exists as methemoglobin (about 1–2 %), which is due to genetic factors or chemical exposure and typically does not result in health issues. When that percentage increases, methemoglobinemia may result, decreasing the oxygen carrying capacity and distribution by the affected hemoglobin. Nitrate reduced to nitrite will oxidize ferrous iron causing a shift to methemoglobin which has been linked to methemoglobinemia in infants whose formula was made with well water [1]. Not all infants developed methemoglobinemia and so it is believed that the involvement of nitrate or nitrite is a cofactor in this disease [2].

Correlations between nitrate intake and gestational complications such as increased spontaneous abortions have been noted [3]. Studies in animals have shown that nitrate, nitrite and *N*-nitroso compounds are all capable of crossing the placenta [4–6], and there have been cases of abortions in pigs pastured or housed in feedlots with oats and rape containing high levels of nitrates, 0.53 % and 5.52 % respectively [7]. Studies have considered whether or not nitrate is involved in spontaneous abortions in humans [3, 8]. A study in Bulgaria found that women with high nitrate exposure suffered from one or more complications, with 67 % having anemia, 23 % toxemia, and 33 % spontaneous abortions or premature delivery. Of the population studied only 16 % had a normal pregnancy [9].

Gastric generation of nitrotyrosines from increased nitrite intake have been detectable in vivo and have been found in tissues associated with multiple pathologies: atherosclerosis, myocardial ischemia, septic and distressed lung, inflammatory bowel disease, and amyotrophic lateral sclerosis [10, 11]. In addition, higher rates of neural tube defects have been reported for fetuses of pregnant women who took nitrosatable drugs or drank water with higher levels of nitrate [12].

Humans are exposed to nitrate predominantly through diet with peak plasma concentrations 60 min after ingestion [13]. Some nitrate exposure is obtained from the environment, and additionally (though minimally) through *de novo* synthesis. In the diet consumption is primarily from fruits and vegetables, which comprise 60–80 % of the nitrate ingested [14]. A secondary source of dietary consumption is cured meats. Sodium nitrate and its reduced form sodium nitrite are used by the meat industry to prevent microbial growth (namely *c. botulinum*) and retain the color of preserved and cured meats [14]. Dietary intake is estimated at 50–140 mg/day in Europe and 40–100 mg/day in the United States [15].

*De novo* synthesis of nitrate has been estimated to range from 500 to 1000 μmol/day [16, 17]. In a study where human subjects consumed a diet with slightly less than average nitrate levels, endogenous nitrate was reported at an average of 870 μmol/day [18]. The higher estimate of 1000 μmol/day translates into 62 mg/day, and when combined with estimates of dietary intake [15], the total nitrate exposure could be as high as 200 mg/day in Europe and 160 mg/day in the United States. Another study using ^15^NO_3_^−^ determined that endogenous nitrate production occurred at all levels of ingestion, however at higher levels of intake endogenous production was masked [16]. The level of nitrate intake per day varies depending on age, gender, race/ethnicity, BMI and level of education [19].

Skeletal muscle development in fetuses of pregnant women exposed to high nitrate levels has not been examined. During skeletal muscle development, myoblasts proliferate and fuse to form multinucleated myotubes. Acetylcholine receptors (AChR) will cluster spontaneously but aggregation increases upon exposure to motor neuron derived agrin [20–22], as AChRs become part of the postsynaptic component of the neuromuscular synapse. In addition, the myogenic regulatory factor myogenin activates genes for AChR subunits [23, 24], suggesting that myogenic regulatory factors like myogenin are intricately linked to the development of the postsynaptic component. Exposure to nicotine, caffeine, ethanol, and mercury have been demonstrated to decrease AChR clustering in C2C12 myotubes [25–28], whereas methoxychlor has been demonstrated to decrease myotube formation by slowing myoblast proliferation without affecting AChR clustering [29].

The objective of the current study was to investigate whether sodium nitrate affects skeletal muscle development, specifically the events of myoblast fusion into myotubes and AChR clustering. And if there is an effect, does sodium nitrate mediate that effect by interfering with myogenin or AChR expression. Skeletal muscle cell cultures, such as the C2C12 cell line derived from mouse hindlimb, provide simplified systems for studying development of the postsynaptic component of the neuromuscular synapse [30, 31]. The C2C12 cell culture model has proven useful for asking fundamental questions concerned with muscle development and neuromuscular synapse formation, and is ideal for examining how sodium nitrate might interfere with these developmental events. The results reported here demonstrate that 1 μg/mL sodium nitrate was sufficient to decrease the frequency of agrin-induced AChR clustering without affecting myotube formation. In addition, sodium nitrate decreased myogenin and AChR gene expression in correlation with the agrin-induced AChR clustering data.

## Methods

### Cell culture maintenance

C2C12 myoblasts were derived from mouse hind limb (gift from H. Gordon, University of Arizona) [30, 31], and are commonly used for skeletal muscle cell culture experiments. They are ideal for studying myoblast fusion to form myotubes, and acetylcholine receptor (AChR) clustering. For normal maintenance of C2C12 cell culture, myoblasts were first plated in growth medium (GM) on 10 cm plates at approximately 20 % confluence. GM consists of Dulbecco’s modified Eagle’s medium (DMEM) plus 20 % fetal bovine serum, 0.5 % chick embryo extract and 100 U/mL penicillin. Fresh GM was added daily, and myoblast cultures were split into new plates at approximately 60 % confluence. For formation of myotubes, myoblasts were plated in GM on 22×22 mm cover slips that had been flamed in 200-proof ethanol and placed in 6-well plates. Fresh GM was added daily. After 48 h in GM, myoblast cultures typically reached 90 % confluence, and cultures were then switched to differentiation medium (DM). DM consists of DMEM plus 2 % horse serum and 100 U/mL penicillin. Fresh DM was added daily as myoblasts fused to form myotubes, and cultures were maintained for 72 h in DM. All cultures were exposed to 10 ng/mL agrin (R&D Systems) for the last 16 h of 72 h in DM to induce AChR clustering. Some cultures were exposed to 10 ng/mL–100 μg/mL sodium nitrate (NaNO_3_; Sigma-Aldrich) for the last 48 h of 72 h in DM. A 1 g/mL NaNO_3_ stock solution was prepared in DM and then serial dilutions were performed to achieve the treatment concentrations. It would be difficult to correlate nitrate dietary intake and endogenous nitrate production with nitrate treatment in cell culture. At best cell culture might mirror fetal exposure to toxins in maternal blood to some degree. Therefore, a range of NaNO_3_ concentrations were tested in cell culture to investigate any effect on AChR clustering, myotube formation, and myogenin and AChR gene expression.

### Acetylcholine receptor clustering assay

AChRs were labeled by the binding of α-bungarotoxin conjugated to tetramethyl rhodamine (Molecular Probes) [32]. Cultures were incubated in the toxin-containing medium for 30 min at 37 °C to label AChRs after 72 h in DM. The cover slips were rinsed three times with room temperature phosphate buffered saline (PBS), fixed for 10 min with 2 % paraformaldehyde in PBS, rinsed three times with PBS, dehydrated in cold methanol for 5 min at −20 °C, and mounted on microscope slides in Vectashield Mounting Medium for Fluorescence (Vector Laboratories). For some experiments the mounting medium contained 4’6-diamidino-2-phenylindole (DAPI) to visualize nuclei. AChR clusters were visualized with an IX70 Olympus inverted microscope under the 20X objective (yielding a total magnification of 200X), and fluorescent images were captured as high-resolution JPG files with an Olympus camera with Magnafire digital imaging software. Bright clusters of AChRs were observed on all aspects of myotubes in fluorescent images. AChR clusters were counted from 25 images captured from each cover slip using ImageJ software. One cover slip was analyzed for each treatment group in each replicate experiment. These data were utilized to assay agrin-induced AChR clustering in untreated cultures, or after exposure to 10 ng/mL–100 μg/mL NaNO_3_ for the last 48 h of 72 h in DM. Comparisons of untreated cultures with cultures exposed to NaNO_3_ were analyzed by Student’s *t*-test to determine statistically different results at *p* < 0.05. Representative images were assembled into Fig. 2.

### Myotube formation index

Cell cultures were visualized with an IX70 Olympus inverted microscope under the 20X objective (yielding a total magnification of 200X), and representative phase contrast and DAPI images were captured as high-resolution JPG files with an Olympus camera with Magnafire digital imaging software. Composite images were created through GNU Image Manipulation Program (GIMP 2) which was used to identify myoblasts (defined as cells with one or two nuclei) or myotubes (defined as cells with three or more nuclei). DAPI staining was used to observe and count nuclei. These images were utilized to quantify myotube formation by modifying a myoblast fusion index paradigm [33] into a myotube formation index used in our laboratory [26, 28, 29]. Six composite images were analyzed for untreated cultures, and five composite images each for cultures exposed to 1 μg/mL NaNO_3_ or 10 μg/mL NaNO_3_ for the last 48 h of 72 h in DM. The myotube formation index was then calculated as the fraction of nuclei in myotubes, with the data displayed in Table 1.

**Table 1:** Myotube formation index

	Total nuclei	Nuclei in myotubes	Nuclei in myoblasts	Fraction of nuclei in myotubes
Untreated (*n* = 6)	2440	2177	263	0.8922
1 μg/ml NaNO_3_ (*n* = 5)	2372	2088	284	0.8803
10 μg/ml NaNO_3_ (*n* = 5)	1917	1653	264	0.8623

### Western blots

To assay for protein levels of the myogenic regulatory factor myogenin or AChR, myotube cultures were divided into untreated cultures, and those that had been exposed to 10 ng/mL, 1 μg/mL, or 100 μg/mL NaNO_3_ for the last 48 h of 72 h in DM. Myotube cultures were rinsed twice with calcium- and magnesium-free PBS (CMF-PBS), scraped off in RIPA complete lysis buffer (containing PMSF, sodium orthovanadate, and protease inhibitors), agitated for 30 min on ice, and then centrifuged at 13,000 g for 2 min to create pellets containing insoluble materials such as organelles and extracellular matrix. The extracted supernatant was then frozen. At a later time samples were thawed, a BCA protein assay was performed to determine the concentration of protein in each sample, and samples were boiled for 5 min in sample buffer to reduce and denature proteins. Then samples were separated by electrophoresis on a 10 % polyacrylamide gel (Bio-Rad) and transferred to a nitrocellulose membrane. The membranes were blocked with 5 % milk in TBS-T, with some membranes probed for 16 h at 4 °C with a mouse monoclonal antibody to myogenin (sc-12732; Santa Cruz Biotechnology) at 1:1000 in blocking solution, and then probed by a goat anti-mouse secondary antibody (926–32210; Li-Cor) at 1:10,000 in blocking solution for 30 min. Other membranes were probed for 16 h at 4 °C with a rabbit polyclonal antibody to AChR (SC-11372; Santa Cruz Biotechnology) at 1:1000 in blocking solution, and then probed by a goat anti-rabbit secondary antibody (926–68071; Li-Cor) at 1:10,000 in blocking solution for 30 min. The resultant western blot was then visualized with the Odyssey CLx, a near infrared imaging system. It uses solid state diode lasers to excite at 685 and 785 nm. For scanning, the western blot was placed on the glass surface of the odyssey and covered with a rubber mat. In the Li-Cor software a box was created to define the scanning area of the blot. The 700 and 800 channels were chosen and low quality image quality and 169 μm resolution was checked before “start scan” was pressed. As a loading control, all samples were also probed with a mouse monoclonal antibody to actin (sc-8432; Santa Cruz Biotechnology). The ratio of myogenin or AChR signal to actin signal could then be calculated and compared across treatment groups, along with the percent reduction in this ratio for treatment groups compared to untreated cultures. The western blot data and agrin-induced AChR clustering data is displayed in Table 2.

**Table 2:** Comparison of NaNO_3_ effects

Myogenin expression	Myogenin expression signal	Actin expression signal	Myogenin/Actin ratio	Percent compared with untreated
Untreated	1170	1630	0.7178	
1 μg/ml NaNO_3_	1360	2130	0.6385	89 %
100 μg/ml NaNO_3_	1030	2640	0.3902	54 %
AChR expression	AChR expression signal	Actin expression signal	AChR/Actin ratio	Percent compared with untreated
Untreated	2490	3610	0.6898	
1 μg/ml NaNO_3_	1910	4590	0.4161	60 %
100 μg/ml NaNO_3_	2340	9320	0.2511	36 %
Agrin-induced AChR clustering			AChR clusters per field of view	Percent compared with untreated
Untreated			19.24	
1 μg/ml NaNO_3_			9.14	48 %
100 μg/ml NaNO_3_			7.42	39 %

## Results

### NaNO_3_ decreases the frequency of agrin-induced AChR clusters

**Fig. 1:**
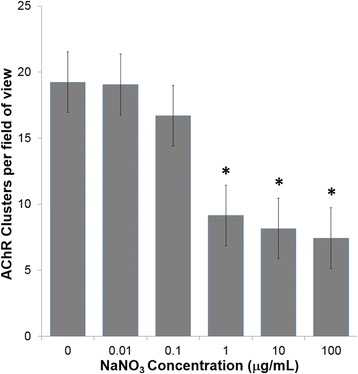
NaNO_3_ decreases the frequency of agrin-induced AChR clusters. Frequency of agrin-induced AChR clusters were quantified by analyzing fluorescent images from agrin-induced cultures that were untreated or were exposed to 10 ng/mL-100 μg/mL NaNO_3_ for the last 48 h of 72 h in DM. The histogram reveals that exposure to 1 μg/mL NaNO_3_ was sufficient to decrease agrin-induced AChR clusters relative to untreated cultures, using Student’s *t*-test at *p* < 0.05. (*) statistically decreased from untreated cultures. Error bar indicates standard error of the mean (s.e.m.)

**Fig. 2:**
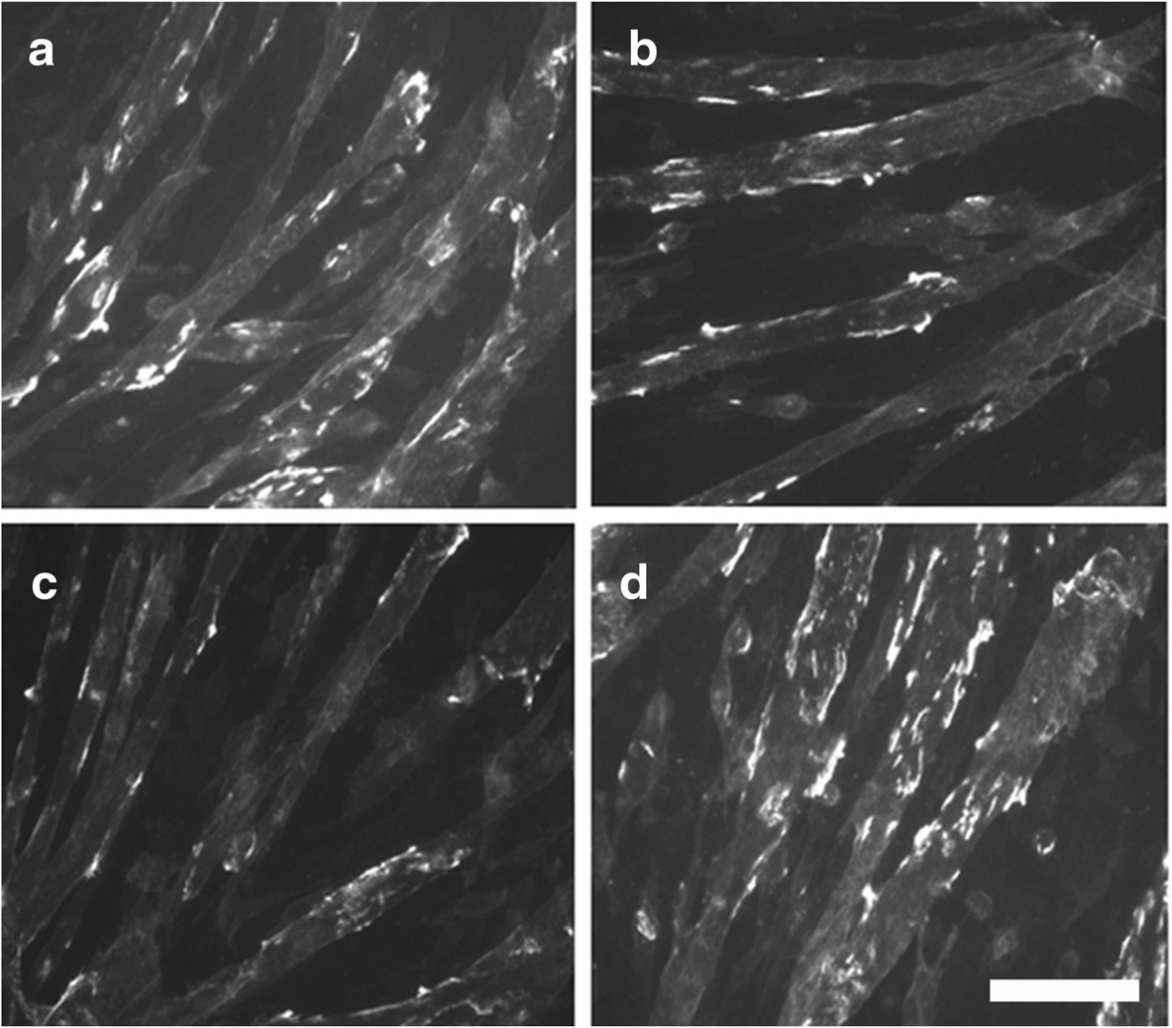
Examples of cell cultures exposed to NaNO_3_. Fluorescent images were captured from agrin-induced cultures that were untreated (**a**), or exposed for the last 48 h of 72 h in DM to 10 μg/mL NaNO_3_ (**b**), 1 μg/mL NaNO_3_ (**c**), or 100 ng/mL NaNO_3_ (**d**). Fluorescent areas are clusters of AChRs. Scale bar = 100 μm

AChRs cluster spontaneously with a baseline frequency on C2C12 myotubes and this clustering is increased with agrin treatment [20–22]. C2C12 cell cultures were switched from GM to DM at 90 % confluence, 10 ng/mL agrin was added for the last 16 h in DM, and myotubes were examined for AChR clustering after 72 h in DM. Some cultures were left untreated (control cultures), while others were exposed to 10 ng/mL–100 μg/mL NaNO_3_ for the last 48 h of 72 h in DM. For each experiment one cover slip was analyzed for each treatment group or control. AChR clusters were counted from 25 images captured from each cover slip using ImageJ software. One cover slip was analyzed for each treatment group in each replicate experiment, with typical data sets reported (Figs. 1 and 2). 1 μg/mL NaNO_3_ was sufficient to decrease the frequency of agrin-induced AChR clustering, with statistical significance at *p* < 0.05 using Student’s *t*-test.

### NaNO_3_ has no effect on myotube formation

**Fig. 3:**
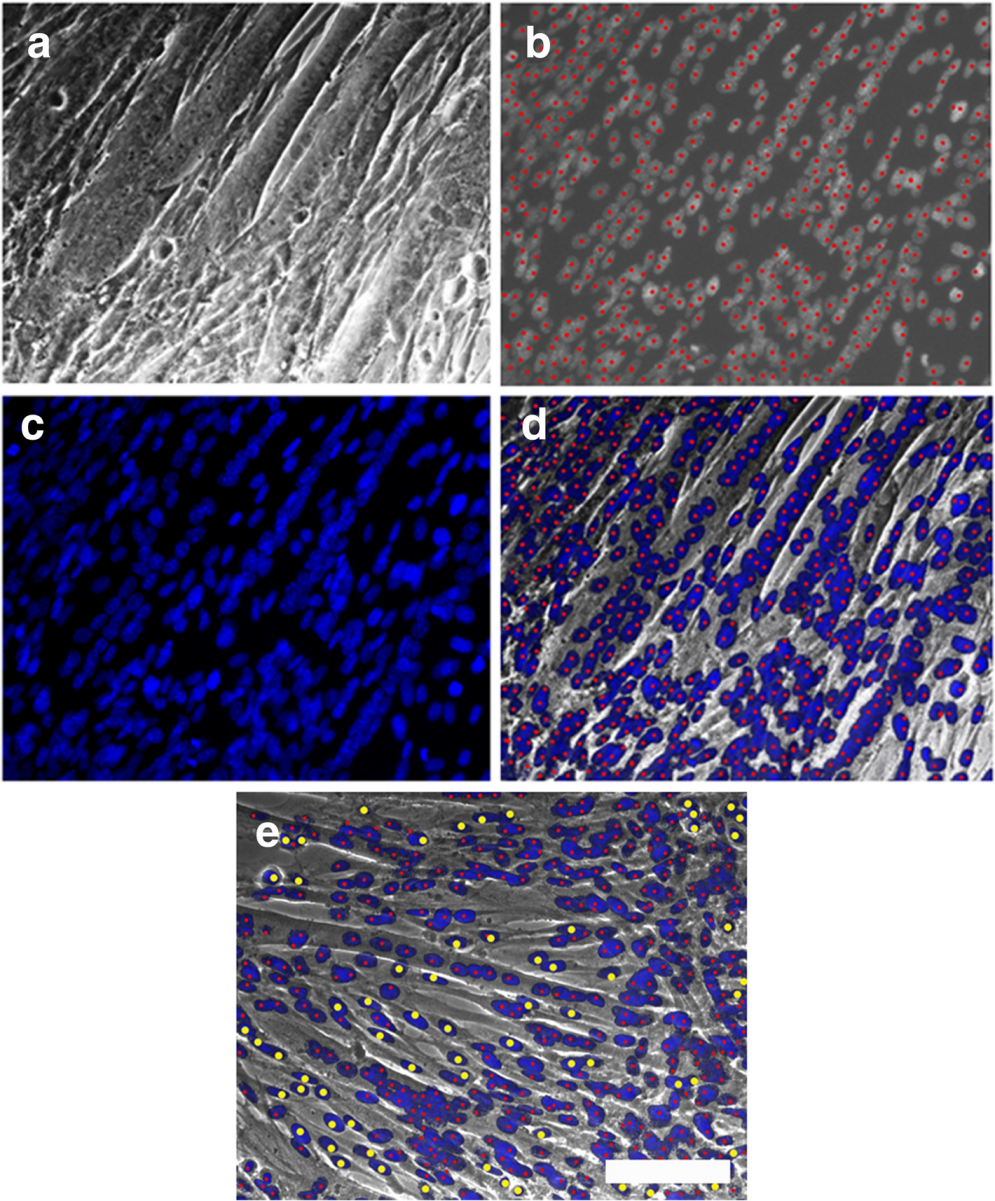
Composite images were created through GNU Image Manipulation Program (GIMP 2) which was used to identify myoblasts (defined as cells with one or two nuclei) or myotubes (defined as cells with three or more nuclei). Phase contrast images were captured to visualize myotubes and myoblasts (**a**), while fluorescent images were captured to label nuclei with dots (**b**) and visualize nuclei with DAPI (**c**). These layers were combined into a composite image (**d**). An example of a composite image with nuclei in myoblasts labeled with yellow (*large*) dots and nuclei in myotubes labeled with red (*small*) dots to allow for quantification of fraction of nuclei in myotubes is included (**e**). Scale bar = 100 μm

Myotube formation was quantified by modifying a myoblast fusion index paradigm [33] into a myotube formation index used in our laboratory [26, 28, 29]. Composite images were created through GNU Image Manipulation Program (GIMP 2) which was used to identify myoblasts (defined as cells with one or two nuclei) or myotubes (defined as cells with three or more nuclei), and then analyzed as described in the Methods and Fig. 3 legend. The myotube formation index was then calculated as the fraction of nuclei in myotubes and presented in Table 1 as a summary of the data collected. The fraction of nuclei in myotubes was unaffected by exposure to 1 μg/mL NaNO_3_ or 10 μg/mL NaNO_3_ for the last 48 h of 72 h in DM.

### NaNO_3_ decreases myogenin and AChR gene expression

**Fig. 4:**
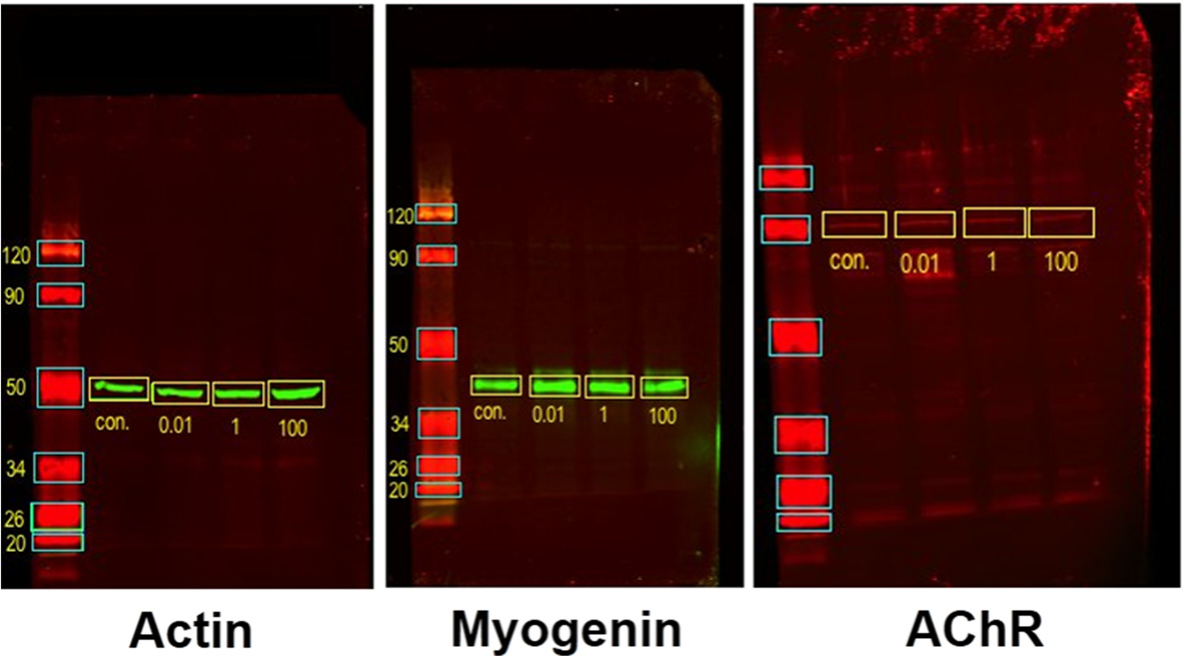
NaNO_3_ decreased myogenin and AChR gene expression in correlation with the agrin-induced AChR clustering data. Untreated agrin-induced cultures were compared with cultures exposed to 10 ng/mL, 1 μg/mL, or 100 μg/mL NaNO_3_ for the last 48 h of 72 h in DM. Cultures were assayed for the amount of myogenin or AChR protein and compared with an actin control. The data correlating these two effects of NaNO_3_ is detailed in Table 2. These results demonstrate that NaNO_3_ decreases the frequency of agrin-induced AChR clustering by a mechanism that causes decreased myogenin and AChR gene expression

The myogenic regulatory factor myogenin activates genes for AChR subunits [23, 24]. This suggests that myogenic regulatory factors like myogenin are intricately linked to the development of the AChR postsynaptic component of the neuromuscular synapse. Therefore a decrease in the frequency of agrin-induced AChR clusters could have many causes. First, NaNO_3_ may decrease AChR gene expression, directly reducing AChR available for agrin-induced clustering of AChRs. Second, NaNO_3_ may decrease myogenin expression, making less myogenin available to activate genes for AChR subunits, indirectly reducing AChR available for agrin-induced clustering of AChRs. Alternately, a decrease in myogenin gene expression could lead to decreased activation of other genes necessary for AChR clustering with or without an effect on AChR gene expression. Finally, NaNO_3_ may decrease AChR clustering through a mechanism that does not affect the level of AChR gene expression. Western blots were used to measure the amount of myogenin protein and AChR protein. C2C12 cell cultures were exposed to 10 ng/mL, 1 μg/mL, or 100 μg/mL NaNO_3_ for the last 48 h of 72 h in DM, with representative western blots presented (Fig. 4). NaNO_3_ decreased myogenin and AChR gene expression in correlation with the agrin-induced AChR clustering data. For the representative experiment in Fig. 4, this correlation is detailed in Table 2.

## Discussion

The results reported here demonstrate that sodium nitrate decreases the frequency of agrin-induced acetylcholine receptor (AChR) clustering in correlation with a decrease in myogenin and AChR gene expression. This suggests that sodium nitrate may interfere with normal developmental events by initially decreasing myogenin gene expression, which causes a decrease in AChR gene expression, and ultimately results in fewer AChRs available to cluster in response to agrin stimulation.

Myogenin is one of several myogenic transcription factors that guide skeletal muscle development. Myogenin directly activates rapsyn gene expression [34] and the genes for AChR subunits [23, 24]. Rapsyn is one of several postsynaptic molecules that is essential for the formation of AChR clusters during neuromuscular synapse formation [35]. Another is a muscle specific kinase (MuSK) that is essential for the signaling events that precede AChR clustering [36–39]. These signaling events are initiated when motor neuron derived agrin binds to low-density lipoprotein receptor-related protein (Lrp4) [40, 41] to stimulate tyrosine phosphorylation of MuSK [38] and the subsequent signaling pathway that includes the tyrosine phosphorylation of the AChR β subunit leading to increased AChR clustering [42, 43]. Suppression of Lrp4 gene expression decreases agrin binding activity, agrin-induced MuSK tyrosine phosphorylation, and agrin-induced AChR clustering [41].

In the data reported in Table 2, when compared with untreated cell cultures, treatment with 1 μg/ml sodium nitrate decreased myogenin gene expression to 89 % of the untreated level, and treatment with 100 μg/ml sodium nitrate decreased myogenin gene expression to 54 % of the untreated level. This correlated with a consequent decrease in AChR gene expression to 60 % of the untreated level with 1 μg/ml sodium nitrate treatment, and a decrease to 36 % of the untreated level with 100 μg/ml sodium nitrate treatment. Decreases in myogenin and AChR gene expression also correlated with decreased agrin-induced AChR clustering, where treatment with 1 μg/ml sodium nitrate decreased agrin-induced AChR clustering to 48 % of the untreated level, and treatment with 100 μg/ml sodium nitrate decreased agrin-induced AChR clustering to 39 % of the untreated level. This data shows that the level of agrin-induced AChR clustering is directly correlated with the level of AChR gene expression, supporting the interpretation that with sodium nitrate treatment fewer AChRs are available to cluster in response to agrin stimulation. Finally, with sodium nitrate treatment of 0.01 μg/ml agrin-induced AChR clustering and myogenin gene expression were unaffected, while AChR gene expression was decreased to 66 % of the untreated level (data not shown), suggesting AChR gene expression is more sensitive to sodium nitrate levels than myogenin gene expression and that AChR gene expression is not the sole determinant of agrin-induced AChR clustering.

Myogenin was previously shown to be essential for myoblast differentiation [44, 45]. In the data reported here, concentrations of sodium nitrate as high as 10 μg/ml had no effect on myotube formation. The fraction of nuclei in myotubes was 0.8922 for untreated cultures, 0.8803 for cultures treated with 1 μg/ml sodium nitrate, and 0.8623 for cultures treated with 10 μg/ml sodium nitrate. In contrast, concentrations as low as 1 μg/ml were sufficient to decrease agrin-induced AChR clustering. This may reflect a difference in sensitivity to myogenin levels between myotube formation and agrin-induced AChR clustering. Alternately, this may reflect the time sequence, since myotube formation occurs prior to synapse formation and AChR clustering in normal skeletal muscle development. A previous study showed that treatment of C2C12 cells with myogenin antibodies also decreased agrin-induced AChR clustering without affecting myotube formation [46].

The reduction in AChR clustering caused by sodium nitrate could have a functional outcome similar to common neuromuscular disorders. As an example, patients with the autoimmune disease myasthenia gravis have muscle weakness due to a decrease in functional AChRs. In contrast, patients with muscular dystrophy have muscle weakness due to neuromuscular synapses that have reduced or absent postsynaptic molecules other than AChRs. However the functional outcome is the same, muscle weakness. The data reported here suggests that any disease process that affects myogenin gene expression could also lead to muscle weakness due to a decrease in AChR clustering at neuromuscular synapses caused by a decrease in AChR gene expression. For further inquiry it might be useful to replicate these experiments using other cell lines including human muscle cell lines to see if the results are universal across species.

## Conclusions

The results reported here demonstrate that 1 μg/mL sodium nitrate was sufficient to decrease the frequency of agrin-induced AChR clustering without affecting myotube formation. In addition, sodium nitrate decreased myogenin and AChR gene expression in correlation with the agrin-induced AChR clustering data. These results demonstrate that sodium nitrate decreases the frequency of agrin-induced AChR clustering by a mechanism that includes myogenin and AChR gene expression. As a consequence sodium nitrate may pose a risk for skeletal muscle development and subsequent neuromuscular synapse formation in humans.
